# The Mechanisms of Chief Executive Officer Characteristics and Corporate Social Responsibility Reporting: Evidence From Chinese-Listed Firms

**DOI:** 10.3389/fpsyg.2022.794258

**Published:** 2022-03-24

**Authors:** Xingxin Zhao, Min Wang, Xinrui Zhan, Yunqing Liu

**Affiliations:** ^1^School of Management and Economics, University of Electronic Science and Technology of China, Chengdu, China; ^2^Guangdong Institute of Electronic Information, University of Electronic Science and Technology of China, Chengdu, China; ^3^Institute of Finance and Public Administration, Anhui University of Finance and Economics, Bengbu, China

**Keywords:** CEO characteristics, CSR report, upper echelons theory, institutional theory, CEO power

## Abstract

Corporate social responsibility (CSR) strategy hinges largely on the CEO characteristics in the context of an emerging market. Based on a sample of 16,144 firm-year observations obtained from 1,370 unique Chinese-listed firms, which whether voluntarily issue CSR reports over the period 2008–2019, this paper empirically examined the impact of CEO characteristics on the likelihood of issuing CSR reports. We find that CEO age, MBA education, international experience and political ideology consciousness are positively associated with the possibility of issuing CSR reports, while a newly appointed CEO will decrease the likelihood of issuing CSR reports. Moreover, we consider a contingent factor, namely CEO power over the board, can significantly enhance the relationship between CEO age, political ideology consciousness, and the likelihood of issuing CSR reports. Furthermore, there’s no significant evidence indicating that CEO power can moderate the relationship between MBA education, international experience, and the likelihood of issuing CSR reports. Nonetheless, CEO power moderates the negative relationship between a newly appointed CEO and CSR reporting initiatives. This study attaches understandings to the extant literature that how top management characteristics can shape firm CSR strategies.

## Introduction

Corporate social responsibility (CSR) has attracted a great deal of attention over the past decade (Jamali and Mirshak, 2007; Aguinis and Glavas, 2012). Various stakeholders such as customers, suppliers, employees, investors, non-government organizations, and social activists have kept asking firms to be more responsible and more transparent (Porter and Kramer, 2006). In response to such stakeholders’ requests, voluntarily reporting CSR information has become a world-wide popular strategic action that firms engage in. On the one hand, studies suggest that the likelihood and quality of CSR report disclosure varies across firms, industries, and regions (Jenkins and Yakovleva, 2006; Peng et al., 2015). On the other hand, some scholars start to recognize that managers might play an important role in explaining the heterogeneity of firms’ CSR participation (Scherer et al., 2016; Nour et al., 2020). They point out that corporate executives are the reason that firms respond to the various stakeholders’ demands or not (Lim and Greenwood, 2017; Clementino and Perkins, 2021). This argument is grounded on the upper echelon theory which posits that firm strategic decisions, including social performance policies, are significantly influenced by corporate executives, in particular, chief executive officers (CEOs) (Tang et al., 2015). Combining current findings, we find that the majority of extant work has focused on the mechanism that CEO’s characteristics on CSR strategies in mature markets, there is lack of understanding of such mechanism on firms’ voluntary CSR reporting in emerging markets.

Therefore, the objective of this study is to examine how CEO characteristics influence firms’ voluntary CSR reporting in emerging markets. China provides us with an ideal research context in the following reasons: first, Chinese firms have witnessed a growing body of firms’ CSR reports in the past decades. Since 2006, China’s central government has issued a series of CSR reporting requirements for selected firms to improve their social and environmental initiatives (Dong et al., 2014; Marquis and Qian, 2014). But many non-target firms started to issue CSR reports as of 2008.

Second, despite the fast growing body of firms’ CSR reports, CSR reporting in China is still its infancy. On the one hand, there is significant variation across listed firms in the quality of CSR reports (Marquis and Qian, 2014). On the other hand, the weak institutional environment and the public CSR awareness lead to highly uncertain costs and benefits of CSR reporting (Zhao et al., 2014). Under such conditions, decisions about firms’ CSR reporting may largely hinge on CEO’s interpretation of government signals. Therefore, CEO plays an important role in determining whether the disclosure of CSR information is perceived as an opportunities or a threat (Jizi et al., 2014). Of course, CEO’s perception and interpretation depends on highly personalized lenses, which are formed by their experiences, personalities, and values (Chin et al., 2013; Maak et al., 2016). Thus, we propose that CEO characteristics play an important role in explaining the variation of firms’ voluntary CSR reporting, especially in the emerging market.

Based on a sample of 16,144 firm-year observations that 1,370 unique Chinese-listed firms whether voluntarily issuing CSR reports from 2008 to 2019, this paper first explores how CEO characteristics, such as age, MBA education, tenure, international experience, and political ideology consciousness, impact the likelihood of issuing CSR reports. Our research findings indicate that the likelihood of voluntary CSR reporting is positively associated with CEO age, MBA education, international experience, and political ideology consciousness. Second, we examine the contingent factor, namely CEO power, that would influence the relationship between CEO characteristics and firm’s voluntary CSR reporting. The results suggest that the CEO’s power over the board can enhance the relationship between CEO age, political ideology consciousness, and the possibility of issuing CSR report. Nevertheless, our results provide evidence on the relationship between CEO tenure, power, and the likelihood of voluntary CSR report.

Our study makes several notable contributions. First, this study shed light on why firms exhibit heterogeneous CSR strategies even when they face similar institutional pressures by addressing CEO’s interpretational role between government signals and firms CSR strategies. Second, it enriches the CSR information disclosure literature by going beyond simply describing the variation of firms’ CSR reporting practices to linking upper echelons theory and providing a better understanding of why firms exhibit different CSR reporting practices.

The remainder of this paper is organized as follows: we reviewed the relevant literature in the next section, and then we proposed our research hypotheses in section “Hypotheses.” The data and methodology are elaborated in section “Data and Methodology.” Section “Results” displays our empirical findings. Finally, we conclude our paper and discussed limitation and future direction in section “Conclusion.”

## Theoretical Background

### Antecedents of Corporate Social Responsibility Reporting

Corporate social responsibility is suggested to be positively related to firm competitive advantages (Porter and Kramer, 2006). Extant literature has revealed the impact of CSR on firm performance in developed countries (Hansen et al., 2011), while the impact of CSR on firm performance is somewhat ambiguous in emerging markets (Bai and Chang, 2015; Ikram et al., 2019). For instance, Wang and Qian (2011) found that corporate philanthropy positively affects the Chinese firms’ financial performance, while Julian and Ofori-dankwa (2013) found that the firm’s long-term operating performance, such as return on sales, return on equity and net profitability, is negatively related to CSR expenditures. And this results in a lack of incentive for firms to implement CSR initiatives.

In China, regulation can be an important antecedent of CSR reporting (Parsa et al., 2021). Since the implementation of reform and opening policy in 1978, the rapid economic growth of China leads to the awakening of people’s awareness of environmental protection (Hart and Milstein, 2003). Subsequently, the central government issued guidelines and recommendations on reporting corporate social and environmental activities (Marquis and Qian, 2014; Farag et al., 2015). Moreover, Shenzhen and Shanghai Stock Exchanges (SSEs) have targeted some specific firms to disclose their CSR practices along with their annual reports from 2008. Therefore, many scholars have concluded that the government is sending signals through many channels that CSR and CSR reporting are appropriate and desire activities (Marquis and Qian, 2014).

Nonetheless, it cannot fully explain firms’ voluntary CSR reporting behaviors. It is important to note that for non-targeted listed firms, these government guidelines and expectations are not mandatory laws (Bown and Crowley, 2013). The government did not specify standards of compliance or penalties for non-compliance. So the CSR reporting of non-targeted firms is done on a voluntarily basis. Previous studies have stressed the importance of corporate characteristics, such as firm size, industry classification, etc. (Cowen et al., 1987; Giannarakis, 2014). Recently, corporate governance has become a growing force to explain voluntary CSR reporting behaviors (Giannarakis, 2014; Jizi et al., 2014). This provides a new lens to study the impact of corporate governance in addition to firm performance (Khatib and Nour, 2021).

### Chief Executive Officer Characteristics and Firms’ Voluntary Corporate Social Responsibility Reporting

Voluntary issuing CSR report is often viewed as a response to market competition, social norms, government regulation, and various stakeholder demands (Tschopp and Nastanski, 2014). Many studies have pointed out the likelihood and quality of CSR report disclosure vastly varies across firms, industries, and regions (Luo et al., 2017; Mani et al., 2018). However, the prior studies fail at the micro-level in explaining how two similar companies facing the same set of environmental constraints can have vastly different levels of CSR reporting.

Some studies have noted that the heterogeneity in corporate’ CSR activities is due to differences among company top leaders (Lockett et al., 2006; Brammer et al., 2012; Wang et al., 2016). For instance, a few studies have examined how CEO’s personal backgrounds and experiences influence corporate CSR participation. Manner (2010) found that firms with female CEOs who have a bachelor’s degree in humanities and a breadth of career experience were more likely to achieve higher CSR performance. Slater and Dixon-Fowler (2009) showed that CEOs with international assignment experience are more likely to lead firms to participate in socially responsible activities. In addition, some recent studies have found the influence of CEOs’ cognitive factors on corporate CSR engagement. For example, Chin et al. (2013) found that CEOs’ political ideologies influenced their firms’ CSR practices: Compared with conservative CEOs, liberal CEOs exhibited greater participation in socially responsible activities.

The above evidence clearly points out the influence of CEO demographic attributes, personalities, and values on firm’s social initiatives. Comparing with mature studies in the western context, there’s still a lack of literature in the merging context (Farag and Mallin, 2018). We bring this line of research into CSR information disclosure research by arguing that CEO characteristics play a significant role on firms issuing CSR reports in the emerging markets. CEO, as a critical member of top management, has discretion on whether to report CSR of the firm. So, CEOs are likely to judge by their own perception and interpretation when deciding upon a CSR information disclosure strategy (Khan et al., 2013). Upper echelon theory indicates that executives’ perception and interpretation of external institutional pressures are influenced by their experiences, capabilities, values, and personalities (Hambrick and Mason, 1984). Therefore, consistent with upper echelon theory, we make hypotheses about how CEO characteristics influence the likelihood of voluntarily issuing CSR reports.

## Hypotheses

### Chief Executive Officer Age

In explaining how CEO age influences CSR initiatives, Donaldson (1999) argued that with increasing age comes the preference for established routine, hesitation to challenge the system of formal rules, and a more deliberate approach to decision making. It seems reasonable to expect that maturity is associated with moral development and firms with older CEOs are more likely to engage in corporate social activities. Outside of moral explanations, older CEOs may be more likely to issue CSR report because older CEOs are more willing to and good at interpreting government signals than younger CEOs. First, aged CEOs have more experience to deal with uncertain and complex institutional environment than younger CEOs (Laufs et al., 2016). So, aged CEOs are able to diagnose the value of government signals more accurately, deeply understand the important role that government plays in firms’ survival and development process, and balance various stakeholder demands. Second, aged CEOs are a little bit more conservative and risk adverse than younger CEOs (Serfling, 2014). Under the conditions of difficultly calculating the cost and the benefits of CSR disclosure, younger CEOs may choose to ignore government signals and lead firms to not issue CSR report. Thus, we hypothesize the following:

**Hypothesis 1**: *Firms led by aged CEOs are more likely to voluntarily issue CSR report than that led by younger CEOs.*

### Chief Executive Officer Education Background

A significant body of research suggests that MBA-educated executives behave differently from executives without MBA degrees (Cannella et al., 2009). It appears that executives with MBA degrees tend to follow more aggressive and innovative strategies and respond to clear-cut and unambiguous environmental changes (Bertrand and Schoar, 2003). Left unanswered in all this is whether the shareholder maximization ethic of MBA-educated executives affects the firm’s attention to other stakeholders, such as customers, employees, communities and governments.

Some scholars point out that executives with MBA degrees are more skilled in strategic decision making and poses a greater capacity to recognize and take advantage of opportunities that would increase firms’ value (Barney et al., 2001). So they can get rich information from many channels use sophisticated techniques and skills to evaluate these information (Graham and Harvey, 2001). So, CEOs with elite MBA degrees might be more likely to perceive government signals on CSR reporting as a strategic opportunity and they want to take this opportunity to enhance firm’s reputation and legitimacy (Carroll and Shabana, 2010). Therefore, the following arguments lead us to offer the following hypothesis:

**Hypothesis 2**: *Firms led by CEOs with MBA education experiences are more likely to voluntarily issue CSR report than that without MBA education experiences.*

### Chief Executive Officer Tenure

A large body of prior study suggests that executives’ tenure in the position, the organization or the industry is highly positively related to strategic persistence, or inversely related to organizational change (Lant et al., 1992; Boeker, 1997; Chowhan et al., 2017). A well-known finding from several studies is that executives tend to make more and bigger strategic changes early in their tenures than they do later on (Cannella et al., 2009). Organizational tenure is thought to be associated with rigidity and commitment to established policies and practices (Lewis et al., 2014). However, newly appointed CEOs have been shown to have fresh and diverse information and are willing to experiment, take risks, and pursue innovative strategies (Shimizu and Hitt, 2004; You et al., 2020).

Based on the above findings, it seems reasonable for us to believe that CEO’s tenure in the organization might influence the firms’ social strategies such as corporate social activities and CSR information disclosure. Lewis et al. (2014) found that newly appointed CEOs (tenure < 3) are more likely to respond to the Carbon Disclosure Project by disclosing their environmental information because newly appointed CEOs are more open-minded and less ingrained in the existing norms of the firm than CEOs with longer tenures. Similarly, we argue that newly appointed CEOs in Chinese-listed firm also have incentives to respond to government signals by leading firms to issue CSR report. First, newly appointed CEOs are like to take risk and adopt innovative practices or strategies, so they are more likely to perceive government signals on CSR reporting as a strategic opportunity which may enhance firms’ reputation or legitimacy. Moreover, long-tenured CEOs have more power over the organization, which allows them to resist pressures from various stakeholders. Therefore, we conclude that newly appointed CEOs are more likely to respond to government signals by leading firms to issue CSR reports.

**Hypothesis 3**: *Firms led by newly appointed CEOs are more likely to voluntarily issue CSR report than that led by long-tenured CEOs.*

### Chief Executive Officer International Experience

The growing body of research has shown that executives’ international experience has been related to higher salaries (Carpenter et al., 2001), greater firm internationalization (Athanassiou and Nigh, 2000) and increased firm financial performance (Daily et al., 2000). Moreover, some studies have found that international experience have largely influenced personal values (Talavera et al., 2018) and provides the CEO with scarce and valuable resources (Collins, 2021). Recently, scholars started to investigate how executives’ international experience influences corporate social activities. For instance, using a sample of 393, CEOs of S&P 500, Slater and Dixon-Fowler (2009) found that CEO international assignment experience is positively related to corporate social performance. We argue that CEOs with international experience is also positively related with the possibility of issuing CSR report in the Chinese context. First, international experience leads to the changes of personal values (Serfling, 2014). Such open-minded and empathic value makes CEOs more care about other stakeholders’ demands, thereby issuing CSR report to connect with various stakeholders. Second, although CSR is still in its infancy in China, CSR in western countries has become a social norm that companies would comply with. Thus, international experience will increase CEOs’ awareness of the importance of sustainable development, CSR, and CSR information disclosure (Slater and Dixon-Fowler, 2009). Therefore, based on the above arguments, we predict that CEOs with international experience are more likely to issue CSR report.

**Hypothesis 4**: *Firms led by CEOs with international experiences are more likely to voluntarily issue CSR report than that without international experiences.*

### Chief Executive Officer Political Ideology Consciousness

Political changes are examined to be strongly related to market change (Jabarin et al., 2019), while political activities are often conducted by firms to shape the government rule-making process (Hillman, 2003). Recently, some researchers have become interested in the political orientations of business leaders as individuals (Maak et al., 2016; Saleh et al., 2020). They argued that executives’ political ideologies is a centrally important construct for considering individuals’ core beliefs and values, can, therefore, be expected to shape organizational outcomes (Hambrick and Mason, 1984). By defining political ideologies as CEOs stance on the conservatism–liberalism dimension, Chin et al. (2013) provide the evidence that liberal CEOs will emphasize CSR more than will conservative CEOs. So it is important to examine whether CEOs’ political ideology or orientation influence firms’ volunteer CSR reporting practices.

However, China only has a party named China Communist party, so we define the concept of CEOs’ political ideology consciousness as to what extent CEOs will recognize and support government. Given that volunteer CSR reporting in China is mainly driven by state guidelines and signals, it seems reasonable to predict that CEOs with higher political ideology consciousness will more likely to respond to government signals. CEOs need to interpret government signals based on their own values and experience. CEOs with higher political ideology consciousness are more likely to trust and support government regulations or expectations. Thus, CEOs with higher political ideology consciousness will more likely to interpret government signals on CSR reporting as strategic opportunities which might provide firms with critical resources and legitimacy than other CEOs. Therefore, based on the above arguments, we predict the following hypothesis.

**Hypothesis 5**: *Firms led by CEOs with higher political ideology consciousness are more likely to voluntarily issue CSR report than that whit lower political ideology consciousness.*

### The Moderating Role of Chief Executive Officer Power Over the Board

So far, we have talked about how different CEOs characteristics affect firm’s volunteer CSR reporting by viewing CEOs as relatively unconstrained in inject their values and preferences into firms’ business decisions. However, the extent how much CEOs exercising their values and preferences into firms’ business decisions is influenced by one important factor-CEO power over the board. It is widely recognized that CEO vary in how much power they possess (Gomez-Mejia et al., 2001; Farag and Mallin, 2016). For instance, many scholars have acknowledged the differences in CEO’s power relative to that of their boards (Lewis et al., 2014). Such differences in CEOs’ power have been found as an important moderator in the relationship between CEOs’ inclinations and firms’ strategic outcomes. For example, using a sample of 249 CEOs, Chin et al. (2013) have found that CEO’s power positively moderates the relationship between CEO’s political ideology and firms’ CSR participation. They argued that CEOs’ power will influence “the degree to which their inclinations are reflected in firms’ decisions.”

Accordingly, we also expect that CEOs will manifest their values and preferences depend on how much power they possess relative to their boards. Specifically, when CEOs have little power, CEO’s inclinations might be only faintly reflected in their companies’ volunteer CSR reporting. However, when CEOs have substantial power, they will hold great power in firms’ decision-making process, and their personal inclinations will be more vividly reflected in firms’ volunteer CSR reporting practice. Therefore, we provide the following hypothesis.

**Hypothesis 6**: *The relationship between CEO’s characteristics and the likelihood of issuing CSR report is enhanced by the CEO’s power. Specifically, the greater a CEO’s power over the board, the stronger the relationship between CEO characteristics and the likelihood of firms’ volunteer CSR reporting.*

## Data and Methodology

### Data and Sample

To construct our sample, we first collected all the firms that are listed on the Shenzhen (SZE) and SSE from 2008 to 2019. As the main goal of this study is to examine the impact of CEO characteristics on voluntary CSR reporting, we marked all the firms whether they have issued the CSR reports. Additionally, to determine whether the firm voluntarily issues its CSR report, we dropped the firms whose CSR reports are mandatorily required by either the SZE or SSE. More specifically, SZE asks the constituent companies of the SZE 100 index to mandatorily disclose CSR reports. SSE asks following three types of firms to disclose CSR information: Companies in the SSE Corporate Governance Section, companies that issue overseas listed foreign shares and financial companies. We dropped these firms and retain only other firms with voluntary CSR reports or no CSR reports. The CSR reporting information is acquired from the China Stock Market and Accounting Research Database (CSMAR), which is a primary source for the financial statements of Chinese-listed firms and is widely used in prior research (Li et al., 2020; Sarfraz et al., 2020).

Next, we downloaded the financial data and CEO characteristics of these public firms from CSMAR as well. We removed the firms that missing the key variables. Finally, we have a sample of 1,370 unique listed firms and 16,144 firm-year observations. As depicted in Table 1, the financial descriptive statistics and industry distribution are in panel A and panel B, respectively. Panel A of Table 1 indicates that the sample firms are different in size and revenue. According to China Securities Regulatory Commission, Panel B of Table 1 suggests that most of the sample firms are from manufacturing industry.

**TABLE 1:** Sample descriptive statistics.

Panel A: Financial data descriptive statistics						
		Mean	Median	*SD*	Min	Max
Total assets (million RMB)		18785.72	4242.91	87517.91	0.05	2,733,190
Total liabilities (million RMB)		11559.14	1987.19	51373.8	–2.03	1,459,350
Operating revenue (million RMB)		12732.95	2248.88	90381.08	0	2,966,193
Equity (million RMB)		7236.62	2043.28	41305.78	–29068.67	1,444,580

**Panel B: Industry distribution**						

**Industry code**	**Description**	**Obs.**	**Industry code**	**Description**		**Obs.**

A	Agriculture, forestry, fishery and animal husbandry	288	K	Real estate		1,204
B	Mining	567	L	Leasing and business service		179
C	Manufacturing	9,474	M	Scientific research and technology services		47
D	Production and supply of electricity, heat, gas and water	778	N	Water conservancy, environment and public facilities management		154
E	Construction	380	O	Residential services		30
F	Wholesaling and retailing	1,182	P	Education		8
G	Transportation, warehousing and postal services	672	Q	Health and social work		11
H	Hotels and Catering	79	R	Culture, sports and entertainment		141
I	Information transmission, software and information technology services	570	S	Comprehensive		335
J	Financial services	45				

*N = 16,144, the standard of industry classification is given by China Securities Regulatory Commission.*

### Measures

#### Dependent Variables

This study mainly focuses on how CEO characteristics influence their decisions to voluntarily issue the CSR report. Thus, the dependent variable is the *voluntary CSR reporting*, which is a dummy variable that takes the value of 1 if a firm issued a CSR report in a given year, and 0 otherwise.

#### Independent Variables

All of the CEO characteristics are collected from the CSMAR database. *AGE* is the age of the CEO in the fiscal year-end. *MBA* is a binary variable that is used to capture the effect of business education. It takes the value of 1 if the CEO has an EMBA or MBA degree and 0 otherwise. *NEW* is also a binary variable. Following Lewis et al. (2014), a new CEO takes the value of 1 if the CEO had been in duty for less than 3 years and 0 otherwise. *OVERSEA* equals to 1 if the CEO has education or working experience aboard and 0 otherwise.

*POLITICAL* is a dichotomous variable to capture CEO’s political ideology consciousness, which is coded as 1 when CEO has the experience of “(vice) Secretary of the Party Committee,” and 0 otherwise. The reasons that CEOs’ experience of “(vice) Secretary of the Party Committee” reflects CEOs’ political ideology consciousness are the following. First, the aim of the Party Committee in firms is to earnestly implement the Communist Party’s guidelines and policies, disseminate the instructions from the various levels of governments, and make sure the firms keep the same pace with the government. Thus, only those who show their faith and loyalty to government can be chosen to be (vice) Secretary of the Party Committee. Second, the working experience of being (vice) Secretary of the Party Committee allows CEOs to gain more understandings about the government and to cultivate the habit to follow and support governments’ policies and expectations. Therefore, CEOs with the experience of “(vice) Secretary of the Party Committee” might have higher political ideology consciousness.

#### Moderating Variables

The moderator in this study is the CEO’s power over the board. *POWER* is a dummy variable that takes the value of 1 if the CEO is the chairman of the board, 0 otherwise.

#### Control Variables

The control variables included in this study are the *Firm Size*, *Firm Age*, *SOE*, *ROA*, and *Stock Exchange*. *Firm Size* is measured as the natural logarithm of the total assets of the firm. *Firm Age* is the monthly age of the firm that is calculated using the difference between the date of the fiscal year-end and the date of the establishment of the firm. We control firm age since older firms may be more inert and less responsive to new policies (Hannan and Freeman, 1984). *SOE* is a dummy variable that reflects the firm’s ownership. It takes the value of 1 if the firm is a state-owned enterprise, and 0 otherwise. *ROA* is the abbreviation for return on assets, which controls for the firm’s financial status. It is calculated using the operating revenue before depreciation divided by the total assets of the firm in the fiscal year end. *Stock Exchange* equals to 1 if the firm is listed on the SSE and 0 for Shenzhen Stock Exchange. Additionally, we include year dummies to absorb the potential time effects. Moreover, after the Chairman Xi came to power in early 2013, the Chinese government proposed lots of environmentally friendly policies to regulate firm behaviors. We use another alternative year dummy which equals to 1 if the year is between 2013 and 2019, and 0 if the year is between 2008 and 2012. Furthermore, we include industry dummies to control for unobservable industry-wide effects, and the industries categories are classified by the China Security Regulatory Commission.

### Model Specification

We used a logistic regression model to estimate how the CEO characteristics impact the likelihood of a firm voluntarily issuing a CSR report. To determine whether this unbalanced panel is suitable for fixed-effect regression, we run the BP-LM test and the Hausman test; the results indicate that the panel data are suitable for pooled regress model. To account for the potential heteroscedasticity and correlation in the error term, we adopted the robust standard errors in the regression analysis. CEO characteristics data were based on the current year, but some control variables including firm financial performance were lagged by 1 year.

## Results

Table 2 presents the descriptive statistics and correlations for all variables. To investigate whether there is potential multicollinearity, we calculated the variance inflation factors (VIFs). The result shows that the maximum VIF is 2.6; the mean VIF is 1.32, far below the rule-of-thumb cutoff of 10. Therefore, the multicollinearity seems not to exist in our model. Table 3 presents the estimates of the likelihood of a firm voluntarily issuing a CSR report.

**TABLE 2:** Variable descriptive statistics and correlations.

	Mean	*SD*	1	2	3	4	5	6	7	8	9	10	11
1. Voluntary CSR report	0.35	0.48	1										
2. *AGE*	49.37	6.09	0.11***	1									
3. *MBA*	0.03	0.18	0.01	−0.06***	1								
4. *NEW*	0.54	0.50	−0.03***	−0.19***	−0.02**	1							
5. *OVERSEA*	0.05	0.22	0.05***	0.00	0.01	−0.02*	1						
6. *POLITICAL*	0.13	0.33	0.04***	0.04***	0.040***	−0.05***	0.03**	1					
7. *POWER*	0.21	0.41	−0.06***	0.10***	0.05***	–0.00	0.06***	0.18***	1				
8. *Firm size*	22.36	1.65	0.46***	0.20***	−0.02*	–0.01	0.07***	0.00	−0.08***	1			
9. *Firm age*	171.71	69.53	0.01	0.15***	−0.07***	0.03**	−0.02*	−0.15***	−0.03***	0.14***	1		
10. *SOE*	0.58	0.49	0.14***	0.13***	−0.11***	0.05***	−0.09***	−0.09***	−0.23***	0.21***	0.12***	1	
11. *ROA*	5.05	57.28	0.02*	0.00	0.00	–0.02	–0.00	0.01	0.00	0.06***	–0.01	0.001	1
12. *Stock exchange*	0.53	0.50	0.15***	0.03***	−0.10***	0.03***	−0.06***	−0.02*	−0.10***	0.16***	0.13***	0.17***	0.00

N = 16,144; *p < 0.05; **p < 0.01; ***p < 0.001. All significant tests are in two-tailed.

**TABLE 3:** Logit regression models predicting the likelihood of volunteer corporate social responsibility (CSR) reporting for Chinese-listed firms.

	Predicted sign	Dependent variable: Voluntary CSR report			
		Model 1	Model 2	Model 3	Model 4
**Independent variables**					
*AGE*	+		0.01**	–0.00	–0.00
			(2.26)	(−0.86)	(−0.71)
*MBA*	+		0.31***	0.38***	0.39***
			(2.98)	(3.00)	(3.07)
*NEW*	+		−0.16***	−0.19***	−0.19***
			(−3.97)	(−4.42)	(−4.46)
*OVERSEA*	+		0.30***	0.26**	0.26**
			(3.41)	(2.37)	(2.41)
*POLITICAL*	+		0.36***	0.25***	0.24***
			(5.87)	(3.33)	(3.18)
**Interaction terms**					
POWER*AGE	+			0.05***	0.05***
				(6.13)	(6.08)
POWER*MBA	+			–0.26	–0.26
				(−1.19)	(−1.19)
POWER*NEW	+			0.22**	0.22**
				(2.21)	(2.21)
POWER*OVERSEA	+			0.18	0.18
				(0.98)	(0.97)
POWER*POLITICAL	+			0.35***	0.34***
				(2.84)	(2.80)
**Control variables**					
*POWER*		−0.13**	−0.21***	−2.87***	−2.84***
		(−2.56)	(−4.08)	(−6.75)	(−6.69)
*Firm size*		0.85***	0.85***	0.85***	0.85***
		(46.73)	(46.20)	(46.23)	(47.12)
*Firm age*		−0.00***	−0.00***	−0.00***	−0.00***
		(−10.41)	(−9.98)	(−10.12)	(−10.16)
*SOE*		0.25***	0.29***	0.30***	0.29***
		(5.86)	(6.68)	(6.86)	(6.72)
*ROA*		0.00	0.00	0.00*	0.00*
		(1.57)	(1.57)	(1.85)	(1.88)
*Stock exchange*		0.47***	0.49***	0.49***	0.49***
		(11.72)	(12.14)	(12.21)	(12.15)
*Year dummies*		Yes	Yes	Yes	0.09*
					(1.80)
*Industry dummies*		Yes	Yes	Yes	Yes
*Constant*		−19.77***	−20.06***	−19.57***	−19.58***
		(−46.95)	(−45.09)	(−43.35)	(−44.13)
*N*		16, 144	16, 141	16, 141	16, 141
*Pseudo R* ^2^		0.20	0.20	0.20	0.20
*Log pseudo likelihood*		–8340.94	–8296.99	–8272.43	–8276.40

t Statistics in parentheses. *p < 0.1, **p < 0.05, ***p < 0.01. All significant tests in two-tailed.

In addition to the moderating variable *POWER*, Model 1 also includes all control variables: *Firm Size, Firm Age, SOE, ROA, Stock Exchange, Year dummies*, and *Industry dummies.* Model 2 test the hypotheses H1–H5 so that the Model 2 includes all independent variables. To investigate hypothesis H6, the model 3 includes all the interaction terms. To determine whether Chairman Xi’s new policies will drive firms to voluntarily issue CSR reports, Model 4 uses the alternative year dummy.

In Model 2, all the coefficients of CEOs’ demographic characteristics are significantly different from zero at least at the 5% level. More specifically, the coefficients of CEO age, MBA education, international experience, and political ideology consciousness are aligned well with our expectation and all of these four coefficients are positively significantly different from zero. These results indicate that an aged CEO, CEOs with MBA education background or with international working or education experience and CEO’s political ideology consciousness are all positively related to the likelihood of voluntary CSR reporting. Therefore, these results provide support to our H1, H2, H4, and H5. However, the coefficient of newly appointed CEO is negative, which is opposite to our prediction, suggesting that a newly appointed CEO is less likely to voluntarily issue CSR reports. Thus our H3 is not supported.

Model 3 and Model 4 use different year dummies but the results are basically the same. In both models, we added the interaction terms and only the coefficients of POWER*AGE, POWER*NEW, and POWER*POLITICAL are significantly different from zero at least at the 5% level. First, the coefficient of interaction term between CEO power and CEO age is positive, which is consistent with our prediction, indicating that the CEO’s power over the board can enhance its relationship between CEO age and the likelihood of CSR reporting. Second, the coefficient of interaction term between CEO power and CEO’s political ideology consciousness is positive, which is consistent with our expectation, also indicating that CEO power can strengthen the relationship between CEO’s political ideology consciousness and the likelihood of issuing CSR reports. Third, there’s no evidence suggesting that CEO power can enhance the relationship between either CEO’s MBA education or CEO’s international experience and the likelihood of issuing CSR reports.

Interestingly, in Model 3 and Model 4, given the positive and significant coefficient of interaction term between CEO power and a newly appointed CEO, the result indicates that a newly appointed CEO, meanwhile a chairman of the board will increase the likelihood of voluntarily issuing the CSR reports. Whereas the coefficient of *NEW* is negative and significant. These contradictory results motivate a plausible explanation: As we hypothesized, a newly appointed CEO indeed can be more risk tolerable and wants to make a difference in their tenure, but this CEO is not powerful enough to fight against the board. This situation gets better until she becomes the chairman of the board, and then CEO can exert their influence on the social strategies of the firm as she wants.

As for control variables, we observed that firm size, firm age, ownership, and stock exchange can strongly impact the firm’s likelihood of issuing CSR repots across four models. In particular, a larger sized firm is more likely to disclose CSR information, a plausible explanation is that large firms are subject to more regulation. Similarly, state-owned firms are generally assumed to bear more social responsibilities, and our result confirms this assumption. Firms listed in the SSE are more likely to issue CSR reports than those listed in the SZE. A plausible explanation is that SSE attaches more importance on mandatory CSR activities and sets higher requirements, while this can positively affect other firms that are listed in SSE. Nonetheless, firm age is negatively associated with the likelihood of CSR reporting, indicating that long-established firms are less willing to issue CSR reports. This phenomenon is strange, especially since Table 2 has depicted a positive correlation between firm age and firm size, ownership, and stock exchange. A plausible explanation is that the long-established firms tend to be old slickers and less likely to be regulated.

In summary, our hypotheses H1, H2, H4, and H5 are supported, H3 is conditionally supported, and H6 is partially supported.

## Conclusion

### Discussion

Drawing from the upper echelons perspective, this paper aims to examine how CEOs’ characteristics are manifested in firms CSR reporting practices. Using the 16,144 firm-year observations from 1,370 unique listed firms during 2008–2019 in China, our results suggest that: (1) CEOs’ demographic characteristics such as age, MBA degrees, international experience, and political ideology consciousness are positively associated with the likelihood of voluntarily issuing CSR reports; (2) a newly appointed CEO is less likely to voluntarily issue CSR reports, but this possibility gets higher when she gets power over the board; (3) CEO’s power over the board will also strongly enhance the relationship between age, political ideology consciousness, and the likelihood of voluntarily issuing CSR reports. We believe these findings offer broader contributions to research on CSR reporting, upper echelons theory, and institutional theory.

First, by examining how CEO characteristics affect firm response to government signals, our study extends the recent research that aims to explain why firms respond differently even when they face similar institutional pressures (Crilly et al., 2012). While a significant body of research has shown that organizational attributes shape the firm’s response to external institutional pressures (Marquis and Qian, 2014). Our results suggest that CEO’s perception and interpretation of institutional pressures play a critical role in explaining various firms’ response strategies. Specifically, the extent to which institutional pressures will exert influence on firms and how firms respond to these institutional pressures depends on how executives especially CEOs perceive and interpret institutional pressures (Lewis et al., 2014). Furthermore, our study particularly highlights the critical role that CEO characteristics play an important role in explaining the variation of firm response strategy to government signals. Although China has gradually transformed from central-planned economy to market economy, the government still remains a critical source of resources and legitimacy. An appropriate response to governmental signals becomes a critical way of acquiring legitimacy from government (Manner, 2010). However, the lack of rule of law and governmental transparency obscures governmental decision processes and priorities (Clementino and Perkins, 2021). Moreover, governmental signals are not specific laws or regulations and there is great uncertainty about the implementation of these government signals (Luo et al., 2017). Thus, firm CEOs have to interpret governmental signals and decide how to respond based on their own knowledge, experiences, and values too.

Second, our study also provides new insights into the antecedents of CSR reporting practices by bringing upper echelons theory into CSR information disclosure research. In demonstrating that a CEO’s characteristics manifested in a company’s CSR profiles and that a CEO’s power amplifies this relationship, the study highlights the broader importance of focusing on within-firm, especially managerial explanations of CSR reporting. A significant body of prior study has examined organizational attributes and institutional drivers of CSR information disclosure, yielding substantial but incomplete insights (Hillman, 2003; Marquis and Qian, 2014). Recently, some scholars have begun to consider that CSR is a subject to managerial discretion that some CEOs pursue, while others-even those facing the same institutional pressures do not (Chin et al., 2013). Our study addresses this call and highlights the important role that CEOs might play in explaining the variation of firms’ CSR reporting practices.

### Limitations and Future Directions

To overcome some limitations that existed in this study, we offer some directions for future research. First, we only examined how CEOs’ demographic characteristics influence firms’ CSR reporting practices. However, upper echelons theory points that top managers’ psychological characteristics are important determinants of firm behavior and outcomes (Hambrick and Mason, 1984; Hambrick, 2007). Future studies should examine how CEOs’ psychological characteristics such as values or personalities affect firms’ CSR reporting practices. The second limitation is this study only focuses on CEOs. Hambrick and Mason (1984) argue that corporate decision-making is a shared activity and studying top management teams rather than CEOs alone, provides better predictions of organizational outcomes. So future studies could consider how the background, experiences, and values of entire top management teams, as well as those of boards of directors, influence firms’ CSR reporting practices, and other CSR activities. Moreover, although the methods in this paper alleviate the endogenous problem to some extent, there may be more suitable methods to deal with the concerns. Future research should continue to find more appropriate tool variables and adopt 2SLS method to deal with this problem. Finally, the conclusions discovered in the study only verified in the Chinese context, we encourage future researches to explore a wider range of research scenarios to broader the theories.

## Data Availability Statement

The raw data supporting the conclusions of this article will be made available by the authors, without undue reservation.

## Author Contributions

YL conceived and designed the research. XxZ wrote and revised the manuscript. MW provided the data and revised the manuscript. XrZ revised the manuscript. XrZ and YL gave guidance throughout the whole research process. All authors contributed to the article and approved the submitted version.

## Conflict of Interest

The authors declare that the research was conducted in the absence of any commercial or financial relationships that could be construed as a potential conflict of interest.

## Publisher’s Note

All claims expressed in this article are solely those of the authors and do not necessarily represent those of their affiliated organizations, or those of the publisher, the editors and the reviewers. Any product that may be evaluated in this article, or claim that may be made by its manufacturer, is not guaranteed or endorsed by the publisher.
